# The Drosophila Zinc Finger Protein Aef1 Colocalizes with Enhancers and Is Involved in the Transcriptional Regulation of Numerous Genes

**DOI:** 10.32607/actanaturae.27556

**Published:** 2025

**Authors:** N. E. Vorobyeva, J. V. Nikolenko, A. N. Krasnov

**Affiliations:** Institute of Gene Biology, Russian Academy of Sciences, Moscow, 119334 Russia; Engelhardt Institute of Molecular Biology, Russian Academy of Sciences, Moscow, 119991 Russia

**Keywords:** Aef1, SAGA, dSWI/SNF, ORC, CBP, H3K27Ac, enhancers

## Abstract

In our previous studies, we demonstrated that the Drosophila zinc finger protein Aef1 interacts with the SAGA DUB module. The Aef1 binding sites colocalize with the SAGA histone acetyltransferase complex and the dSWI/SNF chromatin remodeling complex, as well as the origin recognition complex (ORC). Aef1 predominantly localizes with the promoters of active genes (55% of all sites) and can be involved in transcriptional regulation. In this study, we showed that Aef1 binding sites in Drosophila S2 cells, located outside gene promoters, are nucleosome-depleted regions and colocalize with the SAGA, dSWI/SNF, and ORC complexes. Aef1 binding sites colocalize with the CBP protein and the H3K27Ac histone tag, which is considered to be an active enhancer mark. An RNA-Seq experiment was conducted in Drosophila S2 cells, both normal and with RNA interference targeting the Aef1 protein, to study the role played by the Aef1 protein in transcriptional regulation. The Aef1 protein was shown to affect the transcription of 342 genes, more than half of those (178 genes) containing Aef1 at their promoters or enhancers. Hence, we infer that the Aef1 protein is recruited to both promoters and enhancers and is involved, both directly and indirectly, in the regulation of the transcription of the respective genes.

## INTRODUCTION

Regulation of eukaryotic gene expression is a complex process involving several successive stages of transcription, mRNA processing, mRNA export from the nucleus, translation, and protein folding [1]. Local chromatin structure, gene location relative to functional nuclear compartments, and long-range interactions between cell regulatory elements constitute an additional level in the regulation of genetic processes in the context of the complex architecture of the eukaryotic genome in the 3D space of the cell nucleus [2, 3, 4, 5].

The highly conserved SAGA coactivator complex, chromatin histone modification (acetylation and deubiquitination) being its main function, is composed of more than 20 protein subunits [6]. The SAGA complex subunits interact with various transcriptional activators, thereby recruiting the complex to the promoters of specific genes [7, 8]. There is a degree of synergism between the chromatin remodeling complex and chromatin modifying complex. The SAGA complex has been shown to acetylate the nucleosomes on gene promoters during transcription activation, leading to the recruitment of the dSWI/ SNF chromatin remodeling complex and stimulation of its remodeling activity [9, 10]. The SWI/SNF chromatin remodeling complex and CBP/p300/Nejire acetyltransferase, which is responsible for tagging active enhancers with H3K27Ac, exhibit considerable functional action on the activation of the ecdysonedependent genes *dhr3 *and *hr4 *in S2 cells [11, 12, 13, 14]. Histone H3 acetylation (H3K27Ac) at enhancers was shown to be required for the activation of ecdysonedependent genes [15].

The Drosophila SAGA and SWI/SNF complexes reside within various regulatory elements of the genome, including promoters, where they often colocalize with the origin recognition complex (ORC) [16]. Replication is initiated at numerous sites known as replication origin sites. The ORC composed of six subunits (ORC1–6) is recruited to the replication origins. The ORC binds specific genomic regions [17]; however, the subunits of this complex exhibit no explicit DNA sequence specificity. Therefore, a question arises: which factors are responsible for the positioning of ORCs in the genome?

Our previous studies revealed that the insulator protein Su(Hw) carrying zinc finger domains interacts with the ENY2 protein (a subunit of the SAGA complex) and recruits the SAGA, SWI/SNF, and ORC complexes to Su(Hw)-dependent Drosophila insulators, thus being simultaneously involved in transcriptional regulation and the positioning of replication origins [18, 19, 20, 21, 22]. A hypothesis has been put forward that there also are other proteins carrying zinc finger domains which interact with the Drosophila SAGA complex and function in a similar manner at other regulatory elements of the genome, including promoters. Further experiments have identified four additional proteins having zinc finger domains: CG9890, CG9609, Aef1 (Adult enhancer factor 1), and CG10543 [23, 24, 25, 26, 27]. These proteins also colocalize with the SAGA, ORC, and dSWI/SNF complexes at their binding sites, preferentially at active gene promoters’ sites, and can be involved in transcriptional regulation. As shown previously, the Aef1 protein is recruited to the enhancers of the *adh*, *yp1*, and *yp2 *genes and affects their transcription [28, 29, 30]. Our earlier study [26] revealed that RNA interference targeting the Aef1 protein affects the transcription of several genes. In order to assess the impact of Aef1 on the transcription of all the genes in Drosophila S2 cells, we conducted an RNA-Seq experiment both under normal conditions and upon RNA interference targeting the Aef1 protein.** MATERIALS AND METHODS Cultivation of Schneider 2 (S2) cells. RNA interference** S2 cells were cultured in Schneider’s Insect Medium (Sigma, USA) supplemented with 10% fetal bovine serum (HyClone, USA) at 25°C. The cells were transfected using the Effectene Transfection Reagent (Qiagen, USA), according to the manufacturer’s protocol. Knockdown of the *Aef1 *gene was performed via RNA interference according to a published protocol [22]. The dsRNA corresponding to a fragment of plasmid pBluescript II SK(-) (Stratagene, USA) was used as nonspecific control for RNA interference. The dsRNA for knockdown of the *Aef1 *gene and control was synthesized using the following primers: Aef1, GAATTAATACGACTCACTATAGGGAGAATGATGCATATCAAAAGCCT and GAATTAATACGACTCACTATAGGGAGATCCGGGATGCTCGCTATGT; pBluesciptIISK(-), GAATTAATACGACTCACTATAGGGAGAGTTACATGATCCCCCATG and GAATTAATACGACTCACTATAGGGAGATTTCGCCCCGAAGAACG.

For each RNA interference experiment, 30 μg dsRNA per 10^6^ cells was used. The experiment was conducted in three replicates. RNA was extracted after 5-day incubation.

**RNA-Seq and identification of differentially expressed genes**

RNA-Seq libraries were constructed using a NEBNext Ultra II Directional RNA Library Prep Kit for Illumina (New England Biolabs). The library quality was verified using Bioanalyzer. The libraries were sequenced on an Illumina HiSeq 2000 genome sequencing system. Raw reads in the Fastq format were aligned to the Drosophila genome dmel_r6.54 using the Hisat2 software [31]; adapters had preliminarily been removed in the Atropos software [32]. The “-a” key enabling the search for multiple alignments to be excluded from the analysis was also employed. Only the unique mapped reads were used for further work by analyzing the NH:i tag among the output data in the Hisat2 software. Differentially expressed genes were identified in the CuffDiff2 software [33].

**Enrichment analysis of protein factors at Aef1 binding sites**

The ChIP-Seq profiles of the Aef1, GCN5, OSA, ORC2, H3, CBP, and H3K27Ac proteins obtained earlier [14, 24, 26, 27, 34] were used to study the colocalization of Aef1 binding sites with various protein factors. This analysis and data visualization were performed using the deepTool2 suite [35].

## RESULTS AND DISCUSSION

**The Aef1 binding sites are nucleosomedepleted regions and colocalize with the SAGA, dSWI/SNF, and ORC complexes regardless of the genomic localization**

**Fig. 1:**
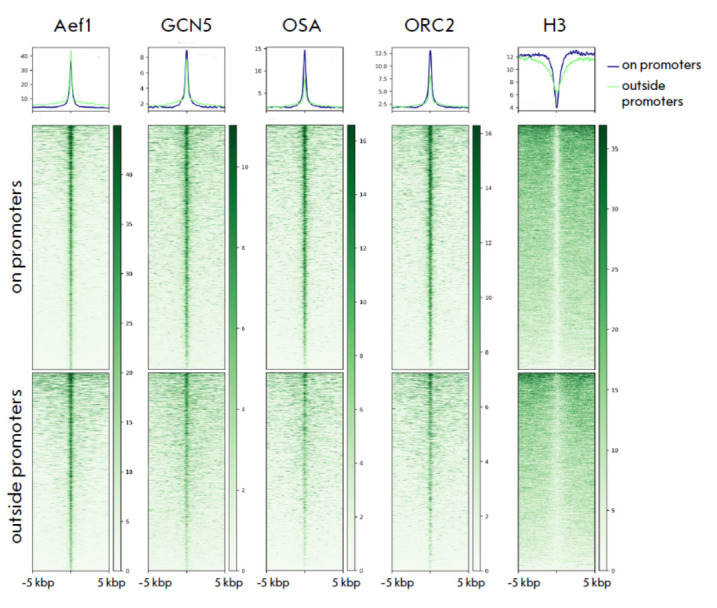
Genomic colocalizations of Aef1 binding sites with GCN5 (the SAGA complex), OSA (the dSWI/SNF complex), ORC2 (the ORC complex), and histone H3. The names of the respective proteins are displayed at the top of the panels. Data are shown for Aef1 sites located in promoters (the middle panel) and outside promoters (the lower panel). The upper panel displays averaged profiles. The blue line represents the profile of proteins located at Aef1 promoter sites, while the green line represents the profile of the proteins on Aef1 sites outside promoters

We have previously demonstrated that the Aef1 protein predominantly resides at the promoters of active genes (55% of sites) and is involved in transcriptional regulation [26]. Aef1 binding sites colocalize with the chromatin modification and chromatin remodeling complexes SAGA and dSWI/SNF, as well as with the ORC replication complex. A considerable portion of Aef1 sites (35%) are located within gene bodies (excluding promoters) and intergenic regions (10%). This study focuses on the properties of the binding sites residing outside promoters. The ChIP-Seq profiles of the proteins Aef1, GCN5 (the SAGA complex), OSA (the SWI/SNF complex), ORC2 (the ORC complex), and histone H3 were utilized for the analysis [14, 24, 26, 27]. We analyzed the enrichment in each of these proteins at two categories of sites: the Aef1 binding sites at promoters and outside them (*Fig. 1*). The results show that the protein complexes under study are recruited to both groups of sites with approximately equal efficiency, although the sites outside the promoters are characterized by lower levels of the OSA and ORC2 proteins. An analysis of the histone H3 distribution revealed that all the Aef1 binding sites were nucleosome-depleted regions, which is typical of active regulatory elements involved in transcriptional regulation [36].

**Aef1 binding sites colocalize with active enhancers**

**Fig. 2:**
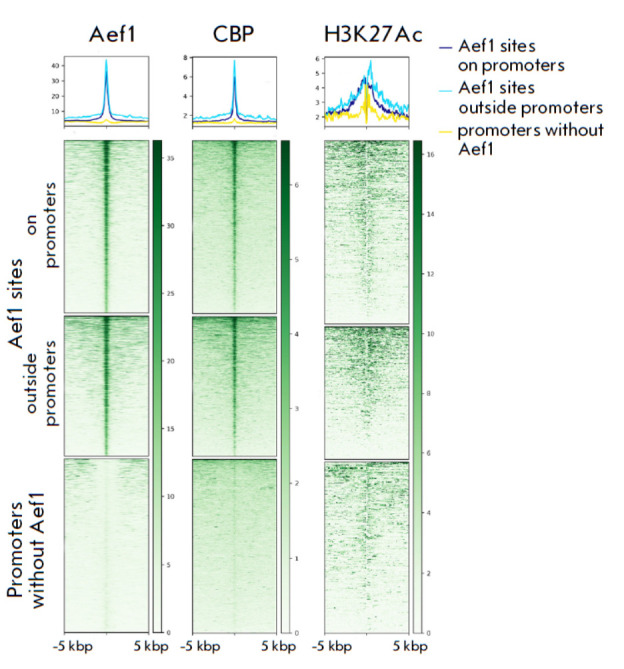
Genomic colocalizations of the Aef1, CBP, and H3K27Ac proteins at three groups of sites: at Aef1 sites on promoters (upper panels); at Aef1 sites outside promoters (middle panels); and at promoters without Aef1 (lower panels). The names of the respective proteins are displayed at the top of the panels

The Aef1 binding sites located outside promoters were analyzed to better understand the nature of the binding sites. Several studies have shown that Aef1 is recruited to enhancers of the *adh*, *yp1*, and *yp2 *genes, affecting their transcription [28, 29, 30]. Histone tag H3K27Ac mediated by acetyltransferase CBP/p300/Nejire is an active enhancer mark. The chromatin remodeling complex SWI/SNF and acetyltransferase CBP/p300/Nejire are recruited to ecdysone-dependent enhancers, which is needed for transcription activation [14]. We aimed to examine the genomic colocalization of the CBP protein and histone tag H3K27Ac at Aef1 binding sites. The previously obtained ChIP-Seq profiles of CBP and H3K27Ac [14, 34] were used. *Figure 2* demonstrates that Aef1 binding sites are characterized by CBP and H3K27Ac recruitment. Therefore, it can be inferred that a significant portion of Aef1 binding sites colocalize with active enhancers.

We noticed that CBP and H3K27Ac also tag the Aef1 binding sites residing in promoters and analyzed another group of sites; namely, the promoters lacking the Aef1 protein (*Fig. 2*, lower panels). An analysis of these sites revealed low CBP and H3K27Ac levels. Hence, recruitment of CBP and the high-intensity H3K27Ac signal at Aef1-carrying promoters correlate with the presence of an Aef1 binding site rather than with the promoter in general.

**Fig. 3:**
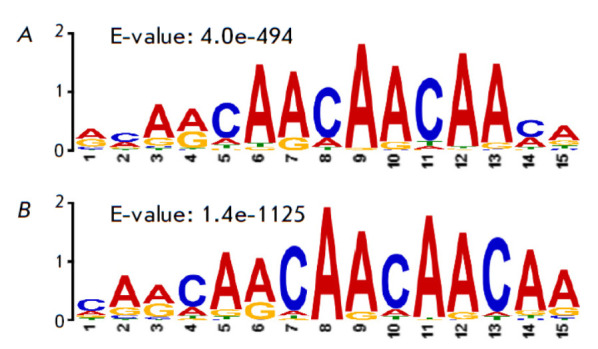
The potential consensus binding motif of the Aef1 protein identified at promoter sites (A) and outside promoters (B). The E-value shows the statistical significance of the result and represents the probability of a random match

We decided to identify the potential consensus motif responsible for Aef1 binding at promoters and outside them using the MEME-ChIP software. An identical consensus motif (CAA)n was identified in both groups of sites (*Fig. 3*), which had been identified previously across the entire set of sites [26], suggesting that both groups of sites appear owing to the DNA-binding properties of Aef1 *per se *rather than via looping of other regulatory elements. These findings are consistent with the data showing that an experimentally confirmed Aef1 binding site within the* adh *gene enhancer contains the CAACAA sequence.

**The Aef1 protein is involved in transcriptional regulation**

As mentioned earlier, Aef1 binding sites are localized both within and outside gene promoter regions. Both groups of sites colocalize with active enhancer marks, indicating that the Aef1 protein could play a role in transcriptional regulation. For this purpose, we conducted a RNA-Seq analysis on Drosophila S2 cells (under normal conditions and with RNA interference targeting the Aef1 protein). The analysis identified 342 genes whose expression was significantly altered (q-value < 0.05) upon RNA interference targeting Aef1. All the genes affected by RNA interference targeting the Aef1 protein were categorized into several groups, depending on whether there were Aef1 binding sites within the gene. It turned out that 57 (17%) genes contained Aef1 exclusively in their promoters, 52 (15%) genes contained Aef1 only in potential enhancers, and 69 (20%) genes had Aef1 in both promoters and enhancers. A total of 164 (48%) genes lacked Aef1 binding sites. These findings suggest that the Aef1 protein localized in both promoters and enhancers is involved in transcriptional regulation. The results also imply that Aef1 may act either directly or indirectly, since there are no binding sites in half of the genes. It is fair to assume that there may exist looping between Aef1 binding sites (potential enhancers) and distal promoters.

## CONCLUSIONS

This study has demonstrated that the Aef1 zinc finger protein is involved in transcriptional regulation. RNA interference targeting the Aef1 protein affects the transcription of 342 genes in Drosophila S2 cells. Approximately half of these genes carry Aef1 binding sites in neither the promoter region nor the gene’s body, which may be indicative of the indirect mechanisms of transcriptional regulation (e.g., via looping between enhancers and promoters). An analysis of Aef1 binding sites demonstrated that they colocalize with active enhancer marks: CBP protein and histone tag H3K27Ac. It is the general property of Aef1 binding sites that is independent of whether they reside in promoters or in the intergenic regions. Aef1-carrying promoters are much more enriched in the CBP protein and H3K27Ac compared to promoters lacking Aef1. That suggests that this property is specific to Aef1 binding sites rather than to promoters in general. It is known that several Drosophila enhancers containing Aef1 reside near transcription start sites (e.g., the *adh *gene enhancer) [30]. It can be hypothesized that Aef1 is a purely enhancer-associated protein, and that its localization at promoters may result from its recruitment to adjacent enhancers.

Our earlier studies [18, 19] have demonstrated that the Su(Hw) protein recruits the SAGA and dSWI/SNF complexes to its binding sites, resulting in the formation of nucleosome-depleted regions and recruitment of the ORC replication complex. Further experiments identified four additional zinc finger proteins (CG9890, CG9609, Aef1, and CG10543) colocalized with the SAGA, SWI/SNF, and ORC complexes [23, 24, 25, 26, 27]. The Su(Hw) protein is predominantly located in intergenic regions at insulators, while the CG9890, CG9609, and CG10543 proteins mainly localize with promoters. Our study has demonstrated that Aef1 binding sites colocalize with active enhancer marks. Despite the differences in genomic localization, all these proteins share properties with respect to the SAGA, SWI/SNF, and ORC complexes. We hypothesize that positioning of the ORC complexes in the genome is regulated by the DNA-binding proteins responsible for the formation of various regulatory elements, including insulators, promoters, and enhancers. We have demonstrated that Aef1 can be an example of such a protein.

## Abbreviations

SAGA: histone acetyltransferase complex
SWI/SNF: chromatin remodeling complex
ORC: origin recognition complex

## References

[1] Orphanides G., Reinberg D. (2002). Cell..

[2] Maksimenko O., Georgiev P. (2014). Front Genet..

[3] van Bemmel J.G., Pagie L., Braunschweig U., Brugman W., Meuleman W., Kerkhoven R.M., van Steensel B. (2010). PLoS One..

[4] Rando O.J., Chang H.Y. (2009). Annu Rev. Biochem..

[5] Tchurikov N.A., Krasnov A.N., Ponomarenko N.A., Golova Y.B., Chernov B.K. (1998). Nucleic Acids Res..

[6] Koutelou E., Hirsch C.L., Dent S.Y. (2010). Curr. Opin Cell Biol..

[7] Baker S.P., Grant P.A. (2007). Oncogene..

[8] Brown C.E., Howe L., Sousa K., Alley S.C., Carrozza M.J., Tan S., Workman J.L. (2001). Science..

[9] Chatterjee N., Sinha D., Lemma-Dechassa M., Tan S., Shogren-Knaak M.A., Bartholomew B. (2011). Nucleic Acids Res..

[10] Li B., Carey M., Workman J.L. (2007). Cell..

[11] Mazina M.Y., Kovalenko E.V., Derevyanko P.K., Nikolenko J.V., Krasnov A.N., Vorobyeva N.E. (2018). Biochim. Biophys. Acta..

[12] Mazina M.Y., Nikolenko J.V., Fursova N.A., Nedil’ko P.N., Krasnov A.N., Vorobyeva N.E. (2015). Cell Cycle..

[13] Mazina M.Y., Kocheryzhkina E.V., Nikolenko J.V., Krasnov A.N., Georgieva S.G., Vorobyeva N.E. (2017). Dokl. Biochem. Biophys..

[14] Krasnov A.N., Evdokimova A.A., Mazina M.Y., Erokhin M., Chetverina D., Vorobyeva N.E. (2023). Int. J. Mol. Sci..

[15] Cheng D., Dong Z., Lin P., Shen G., Xia Q. (2022). Int. J. Mol. Sci..

[16] MacAlpine D.M., Rodriguez H.K., Bell S.P. (2004). Genes Dev..

[17] Eaton M.L., Prinz J.A., MacAlpine H.K., Tretyakov G., Kharchenko P.V., MacAlpine D.M. (2011). Genome Res..

[18] Vorobyeva N.E., Mazina M.U., Golovnin A.K., Kopytova D.V., Gurskiy D.Y., Nabirochkina E.N., Georgieva S.G., Georgiev P.G., Krasnov A.N. (2013). Nucleic Acids Res..

[19] Mazina M., Vorob’eva N.E., Krasnov A.N. (2013). Tsitologiia..

[20] Kurshakova M., Maksimenko O., Golovnin A., Pulina M., Georgieva S., Georgiev P., Krasnov A. (2007). Molecular Cell.

[21] Vorobyeva N.E., Erokhin M., Chetverina D., Krasnov A.N., Mazina M.Y. (2021). Sci. Rep..

[22] Vorobyeva N.E., Krasnov A.N., Erokhin M., Chetverina D., Mazina M. (2024). Epigenetics Chromatin..

[23] Nikolenko J.V., Fursova N.A., Mazina M.Y., Vorobyeva N.E., Krasnov A.N. (2022). Mol. Biol. (Moskow)..

[24] Fursova N.A., Mazina M.Y., Nikolenko J.V., Vorobyova N.E., Krasnov A.N. (2020). Acta Naturae..

[25] Fursova N.A., Nikolenko J.V., Soshnikova N.V., Mazina M.Y., Vorobyova N.E., Krasnov A.N. (2018). Acta Naturae..

[26] Nikolenko J.V., Kurshakova M.M., Kopytova D.V., Vdovina Y.A., Vorobyova N.E., Krasnov A.N. (2024). Mol. Biol. (Moskow)..

[27] Nikolenko J.V., Kurshakova M.M., Kopytova D.V., Vdovina Y.A., Vorobyova N.E., Krasnov A.N. (2024). Mol. Biol. (Moskow)..

[28] An W., Wensink P.C. (1995). Genes Dev..

[29] Brodu V., Mugat B., Fichelson P., Lepesant J.A., Antoniewski C. (2001). Development..

[30] Falb D., Maniatis T. (1992). Genes Dev..

[31] Kim D., Paggi J.M., Park C., Bennett C., Salzberg S.L. (2019). Nat. Biotechnol..

[32] Didion J.P., Martin M., Collins F.S. (2017). Peer. J..

[33] Trapnell C., Hendrickson D.G., Sauvageau M., Goff L., Rinn J.L., Pachter L. (2013). Nat. Biotechnol..

[34] Mazina M.Y., Kovalenko E.V., Vorobyeva N.E. (2021). Sci. Rep..

[35] Ramirez F., Ryan D.P., Gruning B., Bhardwaj V., Kilpert F., Richter A.S., Heyne S., Dundar F., Manke T. (2016). Nucleic Acids Res..

[36] McKay D.J., Lieb J.D. (2013). Dev. Cell..

